# A *de novo* assembly of genomic dataset sequences of the sugar beet root maggot *Tetanops myopaeformis*, TmSBRM_v1.0

**DOI:** 10.1016/j.dib.2024.110298

**Published:** 2024-03-06

**Authors:** Nadim W. Alkharouf, Chenggen Chu, Vincent P. Klink

**Affiliations:** a Department of Computer and Information Sciences, Towson University, Towson, MD 21252, USA; b USDA-ARS-NA- Northern Great Plains Research Laboratory, 1307 N 18TH ST Northern Crop Science Laboratory, Fargo, ND 58102, USA; c USDA-ARS-NEA-BARC, Molecular Plant Pathology Laboratory, Building 004, Room 122, BARC-West, 10300 Baltimore Ave., Beltsville, MD 20705, USA

**Keywords:** Genome, Plant pathogen, Insect, evolution, *Drosophila*, Climate change, Agronomic, Stakeholder

## Abstract

The sugar beet root maggot (SBRM), *Tetanops myopaeformis* (von Röder), is a devastating insect pathogen of sugar beet (SB), *Beta vulgaris*, ssp vulgaris (*B. vulgaris*), an important food crop, while also being one of only two plants globally from which sugar is widely produced, and accounting for 35% of global raw sugar with an annual farm value of $3 billion in the United States alone. SBRM is the most devastating pathogen of sugar beet in North America. The limited natural resistance of *B. vulgaris* necessitates an understanding of the SBRM genome to facilitate generating knowledge of its basic biology, including the interaction between the pathogen and its host(s). Presented is the de novo assembled draft genome sequence of *T. myopaeformis* isolated from field-grown *B. vulgaris* in North Dakota, USA. The SBRM genome sequence TmSBRM_v1.0 will also be valuable for molecular genetic marker development to facilitate host resistance gene identification and knowledge, including SB polygalacturonase inhibiting protein (PGIP), and development of new control strategies for this pathogen, relationship to model genetic organisms like *Drosophila melanogaster* and aid in agronomic improvement of sugar beet for stakeholders while also providing information on the relationship between the SBRM and climate change.

Specifications Table

**Table:** 

Subject	Omics
Specific subject area	Genomics
Data format	Raw, Analyzed, Filtered
Type of data	Table
Data collection	A draft of the *T. myopaeformis* genome was assembled from isolated genomic DNA from a mate-pair library using PacBio Sequel Revio sequencing platform.
Data source location	Fargo, ND, USA
Data accessibility	Repository name: NCBI Data identification number: BioSample accession: SAMN37733483, Temporary SubmissionID: SUB13882507, BioProject ID PRJNA1026092. Direct URL to data: https://www.ncbi.nlm.nih.gov/bioproject/PRJNA1026092 The data has been deposited in Genbank SRA archive found at NCBI under embargo. The data met their requirements for submission. The BioSample accession, Temporary SubmissionID, and BioProject ID that relates to the data are provided above.

## 1 Value of the Data

• The DNA sequence reads provide data that researchers can use to understand insect evolution, the evolution of a pathological niche, host selection, ecology, climate change, and other facets to improve an understanding of their biology and agronomic impact(s).
• The data, deposited in a public database, are available freely for use.
• The data are anticipated to be used for scientific uses. The genomic data can be used to identify targets for the suppression of essential pathogen gene function through RNA interference, mutagenesis, or gene editing through CRISPR/Cas9-mediated processes that synthetically modify genes [[1], [2], [3], [4], [5]]. The suppression of the function of the essential SBRM genes leads to the death of the pathogen, allowing the plant to grow unimpeded by the, otherwise, detrimental effects of the pathogen which would improve the agronomic value of the crop. The reference genome will also allow for a better scientific investigation of the insect and related insect species. The advantage of having a reference genome has been proven over and over in biological studies with subsequent generations of the genome improving on the original sequence [6]; Hoskins et al. 2014.

## 2 Background

*Tetanops myopaeformis* (von Röder), the sugar beet root maggot (SBRM), is a devastating pathogen of sugar beet (SB), *Beta vulgaris*, ssp vulgaris (*B. vulgaris*) [[1], [6]]. SB is an important food crop, while also being one of only two plants globally from which sugar is widely produced, and accounting for 35% of global raw sugar with an annual farm value of $3 billion in the United States alone [[1], [6]]. SBRM is the most devastating pathogen of sugar beet in North America [[1], [6]]. Agricultural control of SBRM is limited by a scarcity of genetic knowledge. The analysis presented here is aimed at providing such a resource for the scientific community and a basis for future updates. The work describes the data, explains its utility to the community, provides protocols and references, and provides a documented link to the data that is in a standard, re-useable format.

## 3 Data Description

A draft *de novo* assembly of the *T. myopaeformis* genome was made, BioSample accession: SAMN37733483, Temporary SubmissionID: SUB13882507, BioProject ID PRJNA1026092. The data are at the URL: https://www.ncbi.nlm.nih.gov/bioproject/PRJNA1026092. PacBio HiFi reads were assembled using the pipeline Flye, version 2.9.2, [2]. Default values were used, except for setting the –asm-coverage argument to 50, to reduce memory consumption. Flye was installed and run on the Windows Subsystem for Linux (Ubuntu 22.04) running on a Windows 2022 workstation with 45 GB of memory. The *T. myopaeformis* sequencing and assembly statistics are summarized (Table 1). The mate-pair library produced a total number of raw reads of 6,356,906. The total read length was 71,844,227,661 and the N50/N90 reads were 11313 and 8294, respectfully (Table 1). The assembly statistics showed a total length of 414,327,873 with the number of contigs of 8,228 (Table 1). The contigs N50 was 57,402. The largest contig was 573,329 bp (Table 1). The mean genome coverage was 94x (Table 1).

**Table 1:** *T. myopaeformis* TmSBRM_v1.0 genome assembly statistics.

Sequencing technology:	PacBio HiFi
Total number of raw reads:	6,356,906
Total read length:	71,844,227,661
Reads N50/N90:	11,313 / 8,294
GC%	40.2
**Assembly statistics:**	
Total length:	414,327,873
Number of contigs	8,228
Contigs N50	57,402
Largest contig	573,329
Mean coverage	94x

## 4 Experimental Design, Materials and Methods

Five larvae of *T. myopaeformis* were collected at Fargo, North Dakota and used as a source of DNA for the analysis. DNA isolation and sequencing of the agriculturally important *T. myopaeformis* was performed at CD Genomics using their proprietary methods. Briefly, DNA was isolated from liquid nitrogen flash frozen larvae. For sample preparation and DNA quality control, isolated DNA quality and quantity were assessed using Qubit and Agilent 5200 Fragment Analyzer. For DNA fragmentation, the DNA was cut into 15 Kb or larger fragments using the Covanis g-TUBE, and subsequently purified using magnetic beads. For DNA repair and end modification, DNA repair enzyme was used to correct any DNA damage and ensure uniformity. Furthermore, the overhang adapters were ligated to the end of the repaired DNA ends, followed by purification using magnetic beads. For SMRTbell library preparation, the repaired and adapter-ligated DNA fragments were then converted into SMRTbell libraries. For SMRTbell library size selection, the SMRTbell libraries were subjected to BluePippin size selection to enrich the fragments over 9-13 Kb and 15 Kb. For binding to polymerase, the size-selected SMRTbell libraries were bound to DNA polymerase molecules. For DNA sequencing, the prepared SMRTbell libraries, with bound polymerase molecules, were loaded onto the PacBio Sequel Revio sequencing platform with options enabled to retain subreads and kinetics sequencing metrics. Furthermore, BAM files generated through HiFi sequencing were converted to FastQ files using the SamTools Fastq algorithm. The FastQ files were imported for genome assembly.

## Limitations

Insects are known to have sex chromosomes. Therefore, the assembly produced here may not totally account for such differences. Consequently, subsequent iterations of SBRM genome sequencing efforts can be done that target this detail.

## Ethics Statement

The authors have read and follow the ethical requirements for publication in Data in Brief and confirming that the current work does not involve human subjects, animal experiments, or any data collected from social media platforms.

## CRediT authorship contribution statement

**Nadim W. Alkharouf:** Methodology, Software, Validation, Formal analysis, Investigation, Resources, Data curation, Writing – original draft. **Chenggen Chu:** Investigation, Resources. **Vincent P. Klink:** Conceptualization, Methodology, Resources, Visualization, Supervision, Project administration, Funding acquisition, Writing – original draft.

## Data Availability

TmSBRM_v1.0 (Original data) (NCBI).
